# Author Correction: Artificial neurovascular network (ANVN) to study the accuracy vs. efficiency trade-off in an energy dependent neural network

**DOI:** 10.1038/s41598-022-11357-8

**Published:** 2022-04-28

**Authors:** Bhadra S. Kumar, Nagavarshini Mayakkannan, N. Sowmya Manojna, V. Srinivasa Chakrava

**Affiliations:** Computational Neuroscience Laboratory, Department of Biotechnology, Indian Institute of Technology Madras, Chennai, India

Correction to: *Scientific Reports* 10.1038/s41598-021-92661-7, published online 05 July 2021

The original version of this Article contained errors in Equation 37, where ${n}_{o}$ was incorrectly given as $n$.

$${{\varvec{R}}{\varvec{M}}{\varvec{S}}{\varvec{E}}}_{{\varvec{c}}{\varvec{o}}{\varvec{n}}{\varvec{t}}{\varvec{r}}{\varvec{o}}{\varvec{l}}\boldsymbol{ }}=\frac{1}{{\varvec{M}}}\sum_{{\varvec{M}}}\sum_{{\varvec{i}}=1:{\varvec{n}}}\frac{{({{\varvec{d}}}_{{\varvec{i}}}\boldsymbol{ }-{{{\varvec{g}}}_{2}({\varvec{h}}}_{{\varvec{i}}}^{{\varvec{s}}}))}^{2}}{{\varvec{n}}}$$

now reads:

$${{\varvec{R}}{\varvec{M}}{\varvec{S}}{\varvec{E}}}_{{\varvec{c}}{\varvec{o}}{\varvec{n}}{\varvec{t}}{\varvec{r}}{\varvec{o}}{\varvec{l}}\boldsymbol{ }}=\frac{1}{{\varvec{M}}}\sum_{{\varvec{M}}}\sum_{{\varvec{i}}=1:{n}_{o}}\frac{{({{\varvec{d}}}_{{\varvec{i}}}\boldsymbol{ }-{{{\varvec{g}}}_{2}({\varvec{h}}}_{{\varvec{i}}}^{{\varvec{s}}}))}^{2}}{{n}_{o}}$$

As a result, in the sentence preceding Equation 37,

“Given a test sample size of M data points, the prediction error was calculated in terms of the root mean squared error (RMSE) between the desired output ${d}_{i}$ and the predicted output ${g(h}_{i}^{s}),$ $1<i<n,$ where $n$ is the number of neurons in the output layer.”

now reads:

“Given a test sample size of M data points, the prediction error was calculated in terms of the root mean squared error (RMSE) between the desired output ${d}_{i}$ and the predicted output ${g(h}_{i}^{s}),$ $1<i<{n}_{o},$ where ${n}_{o}$ is the number of neurons in the output layer.”

Furthermore, Equation 40 contained errors

$${\mathbf{R}\mathbf{M}\mathbf{S}\mathbf{E}}_{{\varvec{k}}}=\frac{1}{{\varvec{M}}}\sum_{{\varvec{M}}}\sum_{{\varvec{i}}=1:{\varvec{I}}}\frac{{({{\varvec{d}}}_{{\varvec{i}}}\boldsymbol{ }-{{\varvec{V}}}_{{\varvec{j}}}^{{\varvec{k}}})}^{2}}{{\varvec{I}}}$$

now reads:

$${\mathbf{R}\mathbf{M}\mathbf{S}\mathbf{E}}_{{\varvec{k}}}=\frac{1}{{\varvec{M}}}\sum_{{\varvec{M}}}\sum_{{\varvec{i}}=1:{n}_{o}}\frac{{({{\varvec{d}}}_{{\varvec{i}}}\boldsymbol{ }-{{{\varvec{g}}}_{2}({\varvec{h}}}_{{\varvec{i}}}^{{\varvec{s}}}))}^{2}}{{n}_{o}}$$

As a result, in the sentence preceding Equation 40,

“The root mean squared error obtained by shutting off $${k}^{th}$$ neuron ($${RMSE}_{k})$$ was calculated as below”

now reads:

“The root mean squared error obtained by shutting off $${k}^{th}$$ neuron ($${RMSE}_{k})$$ was estimated as below by using $${V}_{j}^{k}$$ to estimate the new $${h}_{i}^{s}$$ (see Eq. 10).”

The original Article has been corrected.

