# Supplementary material for: Artificial neurovascular network (ANVN) to study the accuracy vs. efficiency trade-off in an energy dependent neural network
Source: Sci Rep. 2021 Jul 5;11:13808. doi: 10.1038/s41598-021-92661-7 (PMC8257640; doi:10.1038/s41598-021-92661-7)
Supplement: Supplementary file 1 — Supplementary Information. [file 41598_2021_92661_MOESM1_ESM.pdf]

Supplementary material for Artificial Neurovascular Network (ANVN) to Study the Accuracy Vs. Efficiency trade-off in an Energy Dependent Neural Network

Bhadra S Kumar[1], Nagavarshini Mayakkannan[1], N Sowmya Manojna[1], V. Srinivasa Chakravarthy[1*]

1 Computational Neuroscience Laboratory, Department of Biotechnology, Indian Institute of Technology Madras, Chennai, India

* schakra@ee.iitm.ac.in

1. The ANVN with reservoir (ANVN_R) results using EMNIST data set

The ANVN_R was simulated using the EMINST data also. The EMNIST is a dataset similar to MNIST, but with alphabets instead of numbers. We took 500 training data points and 200 test data points for all simulations, such that the data points were equally spanned in 10 predefined classes (Capital Letters A to J). The data was limited to 10 classes so as to use the same network of ANVN_R that we used for MNIST classification.

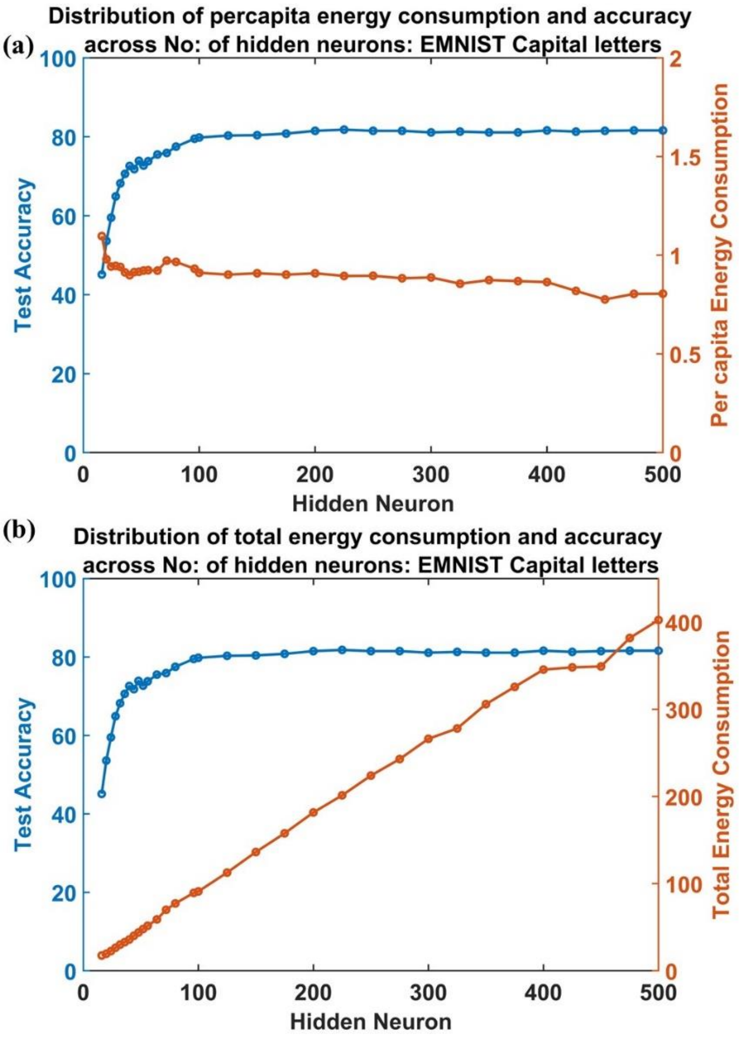

Figure S1: Study of variation in accuracy and energy consumption in ANVN_R with an increase in the number of hidden neurons (using EMNIST data set): (a) Test accuracy vs per capita energy consumption (b) Test accuracy vs total energy consumption

Figure S1.a,b shows the variation of percapita energy and total energy respectively as the number of neurons in the hidden layer increases. Similar to the results of MNIST, the EMNIST also gave a peak efficiency while the number of hidden neurons were around N=28 to N=36 (fig S2.a). Beyond that, the efficiency dropped and accuracy saturated. It was surprising that the robustness of the network to initial energy also was lost (fig S2.b) at almost similar network size (N~64) as obtained when run using MNIST

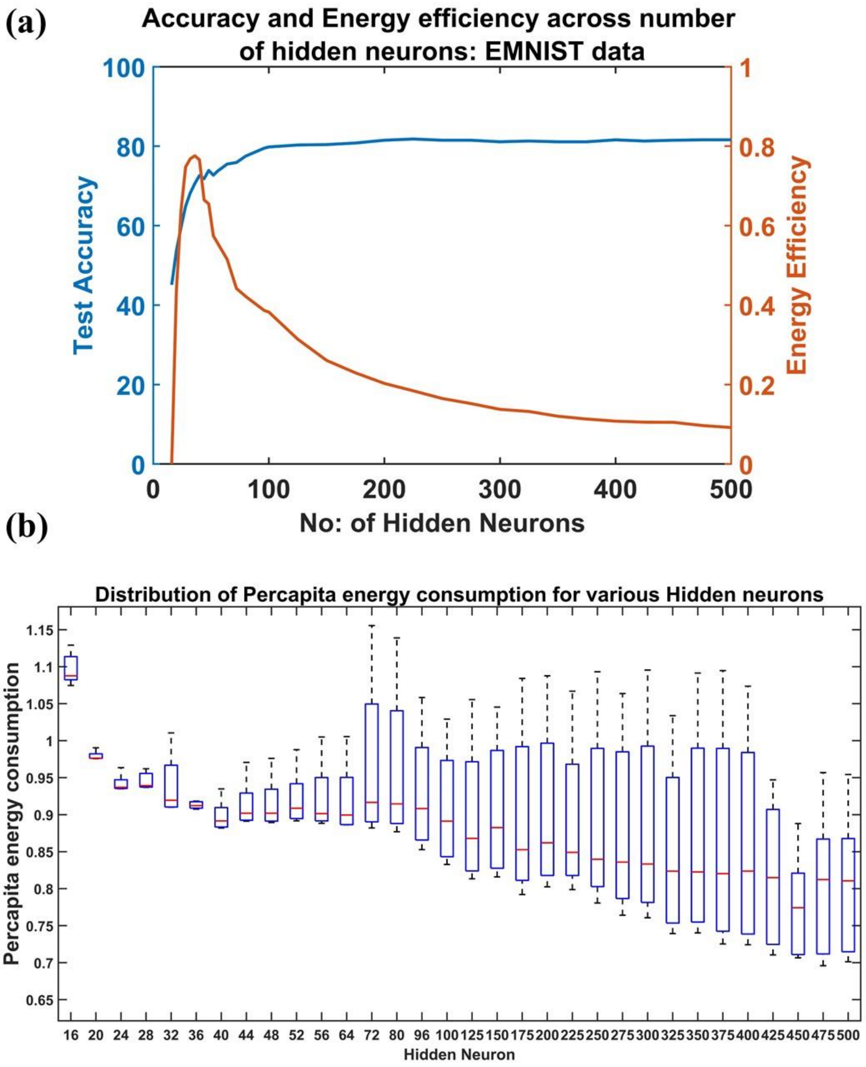

Figure S2: (a) The test accuracy and energy efficiency across the number of hidden layer neurons. (b) The box plot for visualizing the settling points of per capita energy consumption for each initial energy (varied from 0.2 units to 1 unit) given to single neurons given for each network size. The red mark shows the median value of the per capita energy consumed by the trained network and its maximum and the minimum values determine the height of the box (EMNIST data)

2. Transfer learning in ANVN trained under regime 3

The transfer learning was carried out in an ANVN network also and the results were similar to that of ANVN_R. As was observed in ANVN, the network learned faster when second data set was introduced (fig. S.3) and the difference in vascular weights was highest at the level closest to neurons (fig. S4 a,b). (Similar to the results obtained while using MNIST (fig.3 and fig 8.a), in the case of EMNIST also, the ANVN trained using regime 3 gave a lower accuracy than the ANVN with reservoir). The hidden layer had 512 neurons. Input energy given was 500 units. The branching factor was set to k=3 in order to simulate a network with 7 layers.

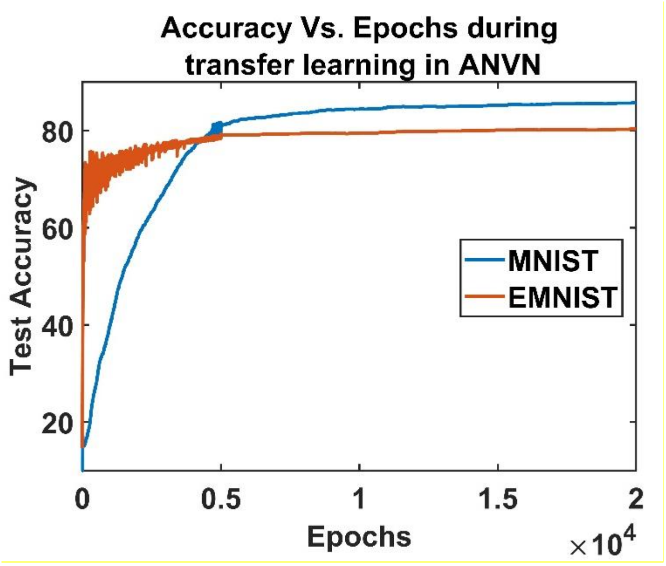

Figure S3: Training accuracy of the initially trained ANVN network (blue) takes more epochs to reach a high accuracy as compared to the network trained by transfer learning (red)

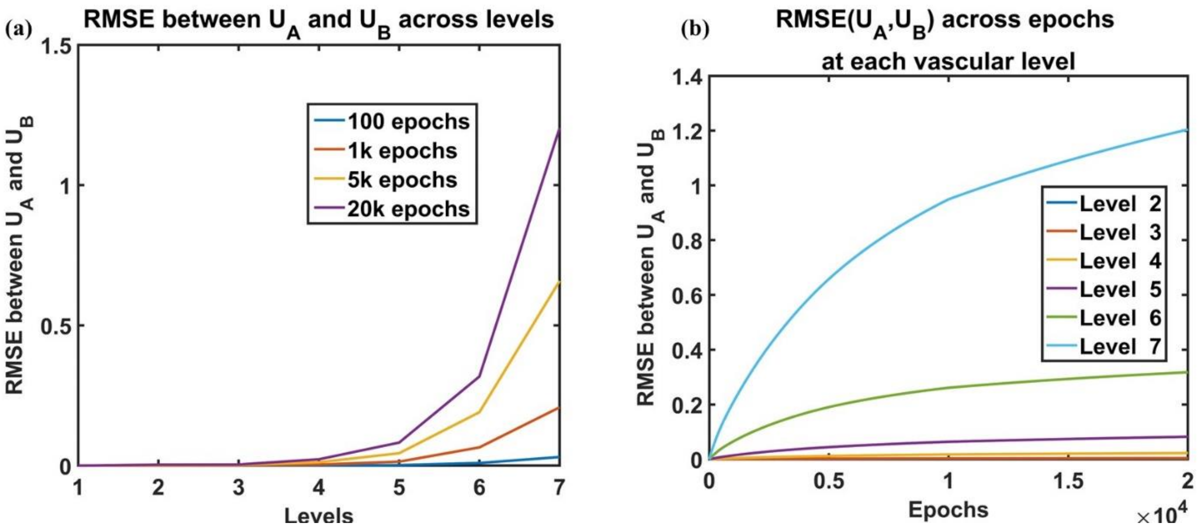

Figure S4: Variation of RMSE of the vascular weight at each level in the vascular tree (ANVN_R) (a) The RMSE between vascular weights at each level. The colors represent the RMSE at specified epochs. (b) The variation of RMSE across epochs for each level

3. The definition of sigmoid function

In the design of ANVN discussed in the main manuscript, the net input at each neuron in the hidden layer is passed through a sigmoid function as described in eqn.9 and eqn.11. A sigmoid function (g) is defined as,

$$\boldsymbol{g(x; a, c) = \frac{1}{1 + e^{-a(x-c)}}} \qquad \textbf{(S.1)}$$

The sigmoid function applied at the hidden layer of the ANVN (g_1) is defined such that $a = 5$ and $c = 0$.

$$\boldsymbol{g_1(x) = \frac{1}{1 + e^{-5x}}} \qquad \textbf{(S.2)}$$

The sigmoid function applied at the output layer of the ANVN (g_2) is defined such that $a = 1$ and $c = 0$.

$$\boldsymbol{g_2(x) = \frac{1}{1 + e^{-x}}} \qquad \textbf{(S.3)}$$

Such a difference in the definition of sigmoid between the two layers is to accommodate the fact that the afferent weights from input layer to hidden layer are normalized and hence the net input (eqn.8) received by each neuron will be between a minimum of -1 (when $\boldsymbol{b_j^f} = 1$) and a maximum of 2 (when $\boldsymbol{b_j^f} = -1$). Hence the sigmoid function needs to have the entire curve limited in this range (red plot in fig. S5). A sigmoid function with lower slope (a=1, blue plot in fig. S5) would fail the purpose of a non-linear transfer function. The output layer on the other hand receives a net input of value with no specific upper bound, hence the sigmoid curve could have a lower slope in the linear portion of the curve (the domain of the transfer function would be much greater than 2 and hence the sigmoid function with a=1 would serve as a good non-linear transfer function). The gradients (eqn 19-22) were calculated considering this modification in the sigmoid function.

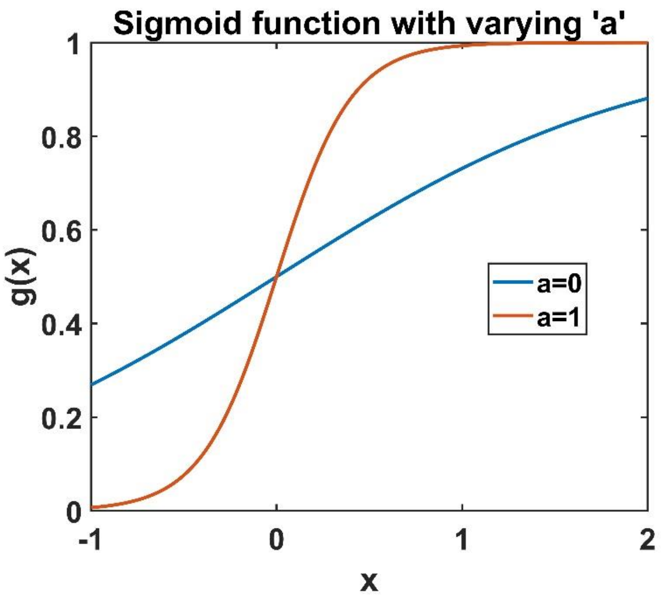

Figure S5: The comparison of sigmoid functions with different values of 'a'.

4. Regularization of Energy

The simultaneous training of neural and vascular networks in ANVN_R showed that increasing the number of hidden neurons beyond a point increases the total energy consumption without much improvement in accuracy. Such behavior might be a consequence of cost function not explicitly demanding minimal energy consumption. Hence, we decided to study the effects of constraining the cost function by introducing regularization. Regularization of weights is a technique widely used to improve the generalization of a neural network[53]. It prevents the overfitting of data. We explored if regularization of energy can bring about any change in the performance of ANVN. Two methods of regularization were explored. One was by penalizing the weights, by implementing L^2 regularization of weights (eqn.**Error! Reference source not found.**). The second method was directly constraining the energy by imposing L^1 regularization of energy (eqn.**Error! Reference source not found.**). For directly including regularization of energy in the cost function, the L^1 regularization of was preferred over L^2 since the biological implication of L^1 minimization of energy was more meaningful. The range of initial values of average per capita energy was varied between 0.4 units and 1 unit. Any neuron receiving a per capita energy > 2 units (due to random initialization of the vascular weights) will return the excess energy by updating the weights using a small negative slope (eqn.**Error! Reference source not found.**). The training data and testing were done using 500 and 200 data points, respectively, from the MNIST data set. Each network was trained for 20k epochs.

The L^2 regularization of weights did not show any notable drop in energy consumption with an increase in the number of hidden neurons (fig. S6.b and S7.b). Instead, the accuracy dropped on introducing L^2 regularization. However, on imposing the constraint directly on the magnitude of the energy consumed by the hidden neurons (L^1 regularization of energy), a significant drop in energy consumption was observed with regularization (Fig S6.a and S7.a). The drop was higher with a higher regularization factor (λ). Moreover, the accuracy was maintained similar to that without regularization. Also, the transition of the network from a fixed-point attractor to a line of attractor appeared to occur much later in the case of a network regularized using L^1 norm of energy (fig. S8.a) when compared to the non-regularized network (fig. 8) and the network regularized using L^2 norm of weights (fig. S8.b). On imposing regularization of energy, the network converges to a fixed-point attractor in the per capita energy consumption vs. accuracy space for a range of larger networks making it more robust to variation in initial energy. Due to the large variation in the accuracy attained by the smallest network across the regularization factors, the relative accuracy, and hence by current definition, the efficiency also cannot be compared across the λ values.

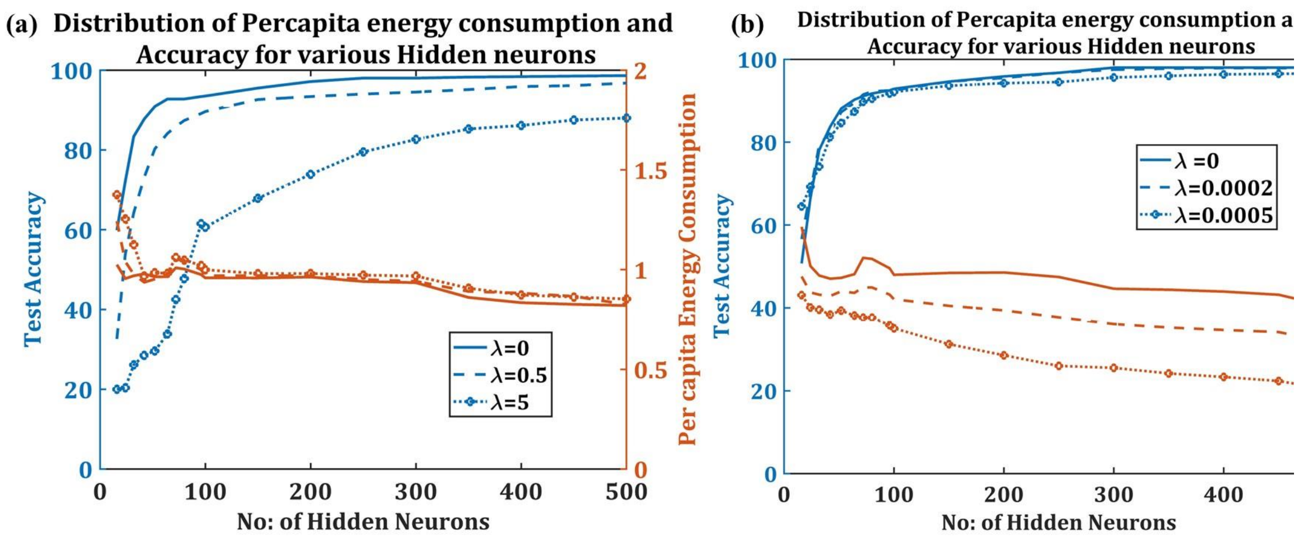

Figure S6: Comparison of performance (Test accuracy vs. Per capita energy consumption) at different values of regularization coefficient, λ in case of (a) L^1 regularization of energy (b) L^2 regularization of weights

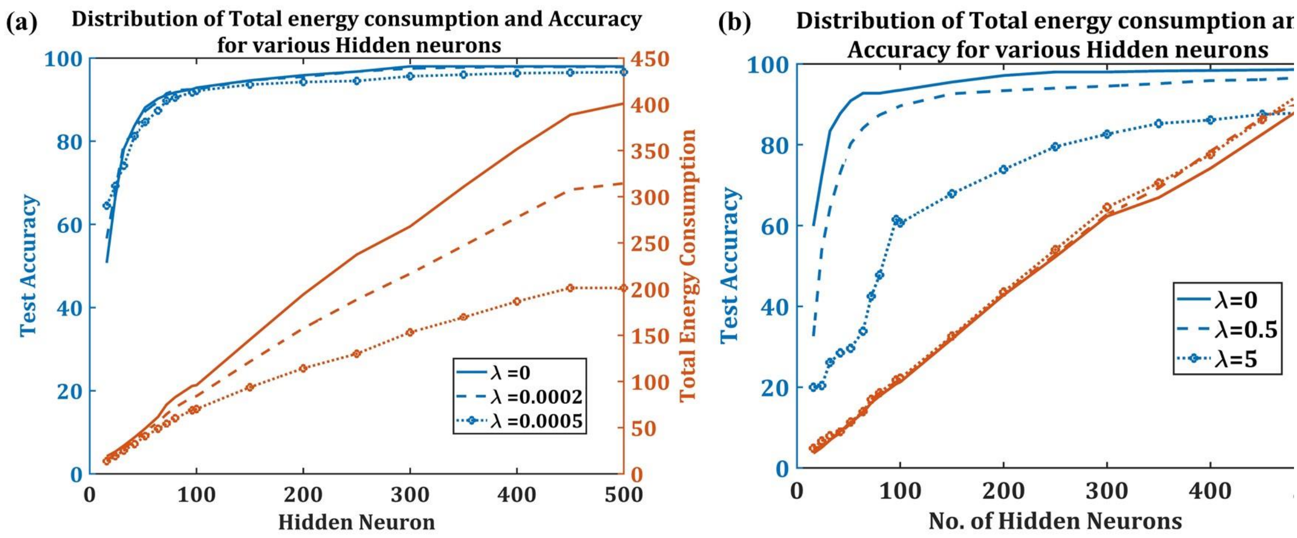

Figure S7: Comparison of performance (Test accuracy vs. Total Energy consumption) at different values of regularization coefficient, λ in case of (a) L^1 regularization of energy (b) L^2 regularization of weights

(a)

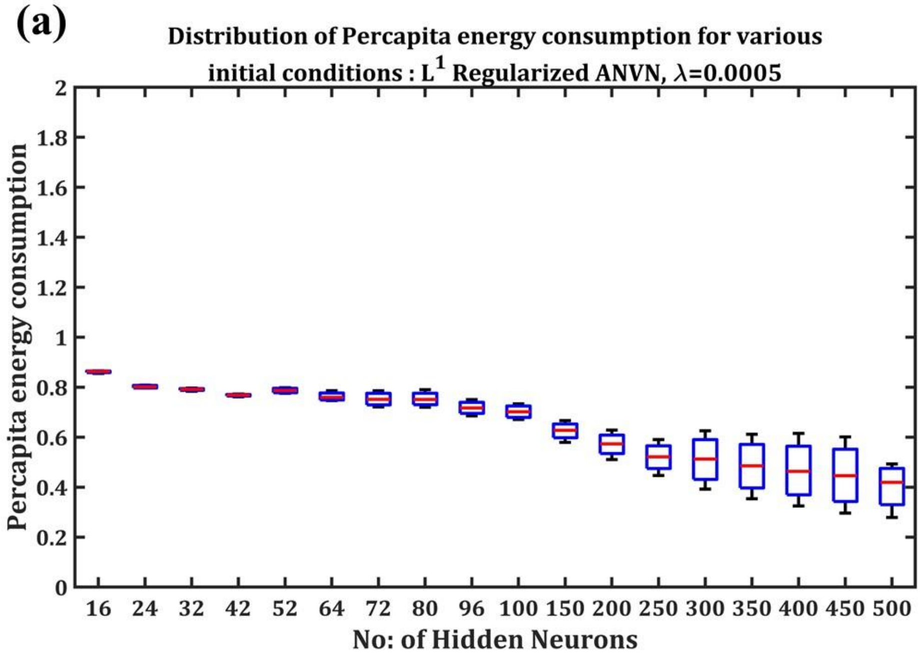

(b)

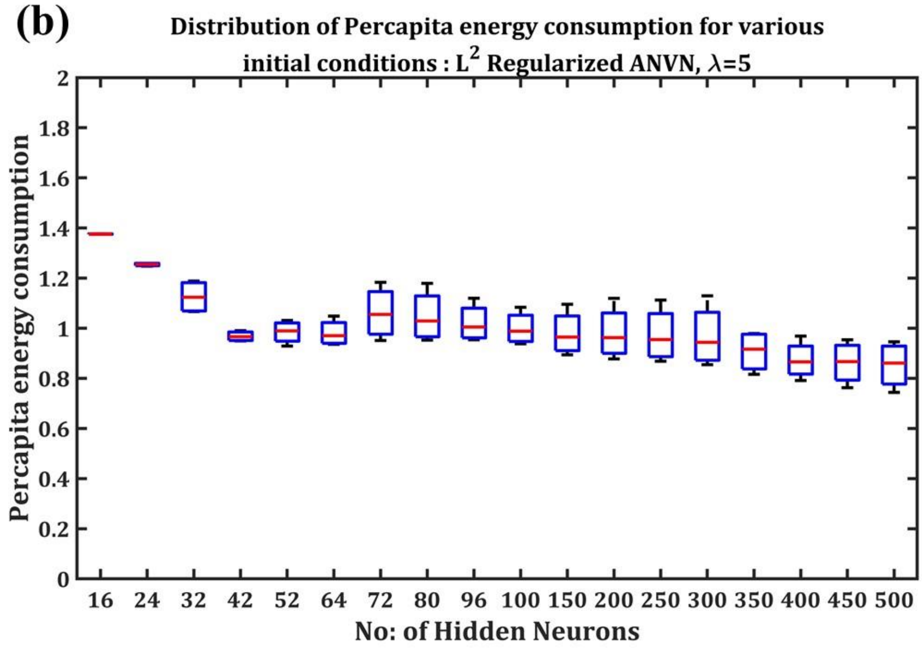

Figure S8: Box plot of variation in percapita energy at different initialization while incorporating (a) L^1 regularization of Energy (b) L^2 regularization of weights

5. Pseudocodes

Pseudocode for untrained ANVN

1. Initialize
    - Neural weights of hidden layer ($\boldsymbol{W}^{f}_{jk}$) and Neural weights of the output layer ($\boldsymbol{W}^{s}_{ij}$) are initialized using the pretrained MLP
    - Vascular weights ($\boldsymbol{U}^{E}_{yx}$) are initialized to ensure equal distribution of energy at each leaf node
2. For Vascular root energy ranging from 10 units to 500 units
    - Compute bias of the hidden layer ($\boldsymbol{b}^{f}_{j}$) using energy at leaf node ($\boldsymbol{E}_{j}$)
    - Compute the output of hidden layer
    - Compute the output of the output layer
    - Compute the accuracy

Pseudocode for sequentially trained ANVN

1. Initialize
    - Neural weights of hidden layer ($\boldsymbol{W}^{f}_{jk}$),Neural weights of the output layer ($\boldsymbol{W}^{s}_{ij}$), and bias of the output layer ($\boldsymbol{b}^{s}_{i}$) are initialized using the pretrained MLP
    - Vascular weights ($\boldsymbol{U}^{E}_{yx}$) are initialized using uniform random distribution
2. For Epochs ranging from 1 to N_{epoch}
    - Compute bias of the hidden layer ($\boldsymbol{b}^{f}_{j}$) using energy at leaf node ($\boldsymbol{E}_{j}$)
    - Compute the difference between the estimated bias and the bias of the trained MLP ($\Delta \boldsymbol{b}^{f}_{j} = \boldsymbol{b}^{f\,MLP}_{j}$ - $\boldsymbol{b}^{f}_{j}$)
    - Compute the energy gradient (ΔE) at the leaf node using $\Delta \boldsymbol{b}^{f}_{j}$
    - Backpropagate the energy gradient along the vascular tree to update the vascular weights from leaf nodes to root node.
3. For Vascular root energy ranging from 10 units to 500 units
    - Compute bias of the hidden layer ($\boldsymbol{b}^{f}_{j}$) using energy at leaf node ($\boldsymbol{E}_{j}$)
    - Compute the output of hidden layer
    - Compute the output of the output layer
    - Compute the accuracy

Pseudocode for simultaneously trained ANVN

1. Initialize
   - Neural weights of hidden layer (W^f_{jk}) and Neural weights of the output layer (W^s_{ij}) are initialized to zeros
   - Biases of the output layer (b^s_i) are random initialized
   - Vascular weights (U^E_{yx}) are initialized using uniform random distribution
2. For Vascular root energy ranging from 10 units to 500 units
   i. For Epochs ranging from 1 to N_{epoch}
      - Compute bias of the hidden layer (b^f_j) using energy at leaf node (E_j)
      - Compute the output of hidden layer
      - Compute the output of the output layer
      - Compute the backpropagation error at the output layer
        - Update the weights and biases of output layer using batch update
      - Compute the backpropagation error at the hidden layer
        - Update the weights using batch update
        - Compute the energy gradient (ΔE) using the gradient of bias (Δb^f_j)
        - Backpropagate the energy gradient along the vascular tree to update the vascular weights from leaf nodes to root node.

   ii. Compute bias of the hidden layer (b^f_j) using energy at leaf node (E_j)
   iii. Compute the output of hidden layer
   iv. Compute the output of the output layer
   v. Compute the accuracy

Pseudocode for simultaneously trained ANVN with reservoir (ANVN_R)

1. Initialize
   - Neural weights of hidden layer (W^f_{jk}) and Neural weights of the output layer (W^s_{ij}) are initialized to zeros
   - Biases of the output layer (b^s_i) are random initialized
   - Vascular weights (U^E_{yx}) are initialized using uniform random distribution
   - Initialize the weights connecting energy source to root node and energy reservoir
2. For network size (no: of neurons in hidden layer) ranging from 16 to 500
   i. For Epochs ranging from 1 to N_{epoch}
      - Compute bias of the hidden layer (b^f_j) using energy at leaf node (E_j)
      - Compute the output of hidden layer
      - Compute the output of the output layer
      - Compute the backpropagation error at the output layer
        - Update the weights and biases of output layer using batch update
      - Compute the backpropagation error at the hidden layer
        - Update the weights using batch update
        - Compute the energy gradient (ΔE) using the gradient of bias (Δb^f_j)
        - Backpropagate the energy gradient along the vascular tree to update the vascular weights from leaf nodes to root node.
        - Update the weight connecting energy source to root node and energy reservoir

   ii. Compute bias of the hidden layer (b^f_j) using energy at leaf node (E_j)

   iii. Compute the output of hidden layer

   iv. Compute the output of the output layer

   v. Compute the accuracy
